# Rives-Stoppa Repair Versus Bilateral Inguinal Hernioplasty: A Comprehensive Review of Surgical Techniques and Patient Outcomes

**DOI:** 10.7759/cureus.65439

**Published:** 2024-07-26

**Authors:** Poosarla Ram Sohan, Chandrashekhar Mahakalkar, Shivani Kshirsagar, Shruthi Bikkumalla, Srinivasa Reddy, Akansha Hatewar, Sparsh Dixit

**Affiliations:** 1 General Surgery, Jawaharlal Nehru Medical College, Datta Meghe Institute of Higher Education and Research, Wardha, IND

**Keywords:** cost-effectiveness, patient outcomes, surgical techniques, bilateral inguinal hernioplasty, rives-stoppa repair, hernia repair surgery

## Abstract

Hernia repair surgery is a common procedure to address the protrusion of organs or tissues through weakened muscles or connective tissue. This review compares two prominent surgical techniques for hernia repair: the Rives-Stoppa repair and bilateral inguinal hernioplasty. The Rives-Stoppa repair involves a posterior approach with extensive mesh placement suitable for complex and recurrent hernias. In contrast, bilateral inguinal hernioplasty focuses on simultaneously repairing bilateral hernias, potentially reducing operative time and enhancing recovery. This review examines each approach's technical aspects, including incision methods, mesh placement, and closure techniques. Patient outcomes, such as postoperative pain, recovery time, recurrence rates, and quality of life, are critically analyzed based on current research and clinical data. Economic considerations are also evaluated, encompassing each technique's cost-effectiveness and economic impact. By synthesizing these findings, this review aims to provide valuable insights for surgeons, healthcare providers, and policymakers in optimizing hernia repair strategies. The evolving landscape of surgical techniques and materials underscores the importance of ongoing research to refine practices and improve outcomes for patients undergoing hernia repair surgery.

## Introduction and background

Hernias occur when an organ or tissue protrudes through a weak spot in the surrounding muscle or connective tissue. This condition can manifest in various body parts, leading to different types of hernias [1]. The most common types include inguinal, femoral, umbilical, incisional, and hiatal hernias. Inguinal hernias, which occur in the groin area, are more prevalent in men due to the natural weakness in the abdominal wall from the inguinal canal. Although less common, femoral hernias appear just below the inguinal ligament and are often found in women [2]. Umbilical hernias near the navel are common in newborns but can also affect adults. Incisional hernias develop at the site of a previous surgical incision, while hiatal hernias involve the stomach pushing up through the diaphragm into the chest cavity. Each type of hernia presents unique challenges and requires specific surgical approaches for effective treatment [3]. Hernias are a widespread health issue globally. Inguinal hernias, for example, affect approximately 27% of men and 3% of women over their lifetimes. Umbilical hernias are seen in 10%-20% of newborns but typically resolve without intervention by three to four years [4]. Femoral hernias are rarer, with a higher incidence in women due to the wider female pelvis. Incisional hernias can develop in up to 15%-20% of patients following abdominal surgery. Hiatal hernias are common in people over 50 years of age. The high prevalence and potential complications of untreated hernias highlight the importance of effective surgical intervention [5].

Surgical repair is the definitive treatment for hernias, aiming to prevent complications such as obstruction, strangulation, and chronic pain. Numerous surgical techniques are available, each with its own advantages and drawbacks. Comparing these techniques is crucial for several reasons [6]. Firstly, patient outcomes can vary significantly depending on the surgical approach, affecting postoperative recovery, recurrence rates, and overall patient satisfaction. Secondly, the cost-effectiveness of surgical procedures impacts healthcare budgets and patient access to care. Finally, ongoing innovations in surgical methods and materials necessitate continuous evaluation to determine the best practices. A comprehensive comparison of surgical techniques can guide surgeons in selecting the most appropriate approach for individual patients, ultimately improving outcomes and resource utilization [7]. This review aims to compare the Rives-Stoppa repair and bilateral inguinal hernioplasty, two widely used techniques for hernia repair. This review will cover technical aspects, including detailed descriptions of each surgical technique, such as incision methods, mesh placement, and closure techniques. Additionally, it will analyze patient outcomes, focusing on postoperative recovery, recurrence rates, long-term complications, and quality of life. Economic considerations will also be evaluated, assessing each technique's cost-effectiveness and economic impact. Finally, the review will explore potential advancements in hernia repair methods and materials. By synthesizing current research and clinical data, this review aims to provide valuable insights for surgeons, healthcare providers, and policymakers, facilitating informed decision-making in hernia repair strategies.

## Review

Overview of Rives-Stoppa repair

Historical Background

The Rives-Stoppa repair technique has a storied history, originating in the 1940s and 1950s with the introduction of metal wire mesh for hernia repair. This technique was later popularised in Europe by surgeons Jean Rives and René Stoppa, who developed the sublay technique for incisional hernia repair [8]. The Rives-Stoppa repair involves placing a prosthetic mesh in a sublay position above the posterior rectus sheath. This approach promotes physiological healing and reduces recurrence rates for inguinal hernias. The use of metal wire mesh for hernia repair in the early 20th century laid the groundwork for this technique's development [8]. Over time, the choice of mesh materials has evolved, with absorbable synthetic, biological, and lightweight mesh becoming more common. These advancements in mesh technology have further improved outcomes and reduced complications associated with the Rives-Stoppa repair [9]. Today, the Rives-Stoppa repair is the gold standard for midline abdominal wall hernias, offering high success rates and low recurrence rates. The technique involves a meticulous dissection of the retrorectus plane and mesh placement in a sublay fashion, facilitating abdominal wall reconstruction and minimizing the risk of recurrence [9].

Surgical Technique

The Rives-Stoppa repair is a well-established surgical technique for treating incisional hernias. The procedure begins with an incision to access the abdominal cavity, followed by the dissection and excision of the hernia sac. A key step in the Rives-Stoppa repair is the dissection of the posterior rectus sheath, which is then closed, allowing for the medial advancement of the rectus abdominis muscles. This posterior component separation is crucial to the technique [10]. Next, a prosthetic mesh, such as a PVDF mesh (e.g., DynaMesh), is placed in the newly created retrorectus space. The mesh is carefully positioned to reinforce the abdominal wall defect. Drains are typically placed during the procedure to help prevent seroma formation. Finally, the anterior rectus sheath is closed over the mesh, completing the layered reconstruction of the abdominal wall [11]. Studies have reported excellent outcomes with the Rives-Stoppa technique, with low rates of early complications (around 8%-9%) and recurrence (around 10%). The use of PVDF mesh, in particular, has demonstrated good outcomes regarding hernia recurrence and surgical site complications. The Rives-Stoppa repair is considered the gold standard for open treatment of midline ventral and incisional hernias due to its ability to achieve a durable, anatomical reconstruction of the abdominal wall. The layered approach, involving posterior rectus sheath dissection and closure, allows for a strong and stable repair that restores the natural anatomy and physiology of the abdominal wall [12].

Indications and Contraindications

**Figure 1:**
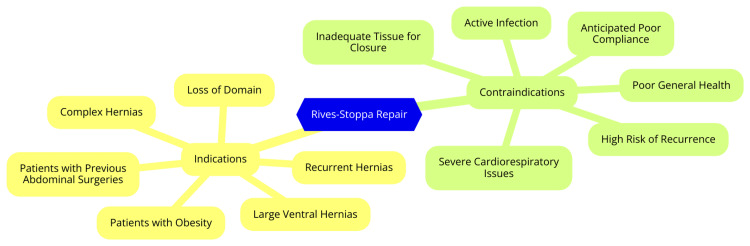
Indications and contraindications of Rives-Stoppa repair Image Credit: Dr. Poosarla Ram Sohan

The Rives-Stoppa repair is a surgical technique to treat midline abdominal wall hernias, particularly incisional hernias. It is considered the gold standard for this type of hernia repair due to its ability to achieve a durable anatomical reconstruction of the abdominal wall [13]. Indications for the Rives-Stoppa repair include midline abdominal wall hernias, large incisional hernias, recurrent hernias, and midline ventral hernias. Patients with these conditions benefit from the technique's strong reinforcement of the abdominal wall and its low recurrence rates [13]. However, there are contraindications to the Rives-Stoppa repair. Patients with severe comorbidities, such as advanced cardiac or pulmonary disease, may not be suitable candidates due to the increased risk of complications and higher recurrence rates. Additionally, patients with severe wound complications, inadequate tissue quality, or a history of multiple prior hernia repairs may require modifications to the technique or alternative approaches [14]. Key steps of the Rives-Stoppa repair include making an incision to access the abdominal cavity, dissecting the posterior rectus sheath, placing a prosthetic mesh in the retromuscular space, and closing the anterior fascia and posterior sheath. This layered reconstruction helps restore the anatomical and physiological properties of the abdominal wall [14]. Studies have reported favorable outcomes with the Rives-Stoppa repair, with recurrence rates ranging from 0% to 8% and manageable complication rates, such as seromas, wound infections, and surgical site events. Specific materials such as PVDF mesh have also demonstrated good results regarding hernia recurrence and surgical site complications [15]. Indications and contraindications of Rives-Stoppa repair are shown in Figure 1.

Overview of bilateral inguinal hernioplasty

Historical Background

One of the earliest recorded interventions for inguinal hernias dates back to 1550 BC, where the Ebers Papyrus describes physicians reducing the hernia and applying heat to the hernia sac. The first surgical inguinal hernia repair was reported by Demetrius Cantemir in 1716, using an open transabdominal approach. Open inguinal hernia repair techniques dominated most of the 20th century [16]. Laparoscopic surgery was first introduced by George Kelling in 1901, but surgical repair of inguinal hernias remained predominantly open for most of the 1900s. In 1982, Ger and colleagues described the first laparoscopic inguinal hernia repair conducted in dogs, using a novel stapling device to close the abdominal opening of the patent processus vaginalis. Bogojavalensky later developed the laparoscopic plug-and-patch repair, which involved inserting a synthetic mesh plug into the hernia defect opening over the peritoneum. This technique fell out of favor due to an increased risk of complications [17]. In 1991, Fitzgibbons et al [18]. described the intraperitoneal onlay mesh (IPOM) repair in pigs using a polypropylene mesh with an adhesion barrier affixed over the peritoneum covering the hernia defect. Later that same year, Toy and Smoot described a similar laparoscopic IPOM technique in ten patients. The introduction of laparoscopic techniques marked the beginning of the evolution towards minimally invasive inguinal hernia repairs, which have continued to develop and improve over time [18].

Surgical Technique

Bilateral inguinal hernia repair can be performed using various surgical techniques, including open and laparoscopic approaches. The Rives-Stoppa repair, also known as the giant prosthetic reinforcement of the visceral sac (GPRVS), is an open tension-free technique that involves a single midline incision to access the preperitoneal space and myopectineal orifice on both sides. This allows for repairing bilateral inguinal hernias through a single incision, avoiding the need for separate incisions. A large prosthetic mesh is then placed to reinforce the entire abdominal wall, and the midline incision is closed with sutures after mesh placement [19]. Another open technique is the Lichtenstein bilateral inguinal hernioplasty, which uses separate incisions on each side to access and repair the inguinal hernias. This involves placing a synthetic mesh over the posterior inguinal wall on each side through the individual incisions, securing the mesh without tension to reinforce the weakened areas and prevent recurrence. The separate incisions on each side are closed with sutures after mesh placement [20]. Laparoscopic approaches, such as total extraperitoneal (TEP) repair, use small incisions to access the preperitoneal space and place a single mesh to reinforce both inguinal regions. This avoids the need for separate incisions, mesh placement on each side, and the use of staple fixation. The small incisions used for trocar placement are closed with sutures after the procedure [21].

Indications and Contraindications

Bilateral inguinal hernia repair is indicated for patients with symptomatic inguinal hernias on both sides, particularly those causing pain, discomfort, or posing a risk of complications such as incarceration or strangulation. Recurrent and large or complex inguinal hernias also warrant bilateral repair [6]. While inguinal hernia repair generally has no absolute contraindications, certain patient factors must be carefully considered. These include unstable medical conditions that increase surgical risk, active infections, and pregnancy, with elective repair typically delayed until at least four weeks after delivery. Patients unable to tolerate general anesthesia or with prior major abdominal surgery that may complicate the surgical approach are also not ideal candidates. Conditions such as ascites or previous pelvic radiation therapy can further complicate laparoscopic approaches [22]. The patient's overall medical status and risk factors must be thoroughly evaluated to determine the most appropriate surgical approach and timing. The benefits of repairing bilateral inguinal hernias must outweigh each patient's potential risks. With careful patient selection and surgical planning, bilateral inguinal hernia repair can be safely and effectively performed using either open or laparoscopic techniques [6].

Comparison of surgical techniques

Technical Complexity

The Rives-Stoppa repair, also known as the GPRVS, is a tension-free technique for abdominal hernia repair that is considered more technically complex compared to bilateral inguinal hernioplasty using the Lichtenstein technique. This approach involves a wide dissection of the preperitoneal space to access the myopectineal orifice and the placement of a large prosthetic mesh to reinforce the entire abdominal wall [19]. The Rives-Stoppa repair allows for the repair of bilateral inguinal hernias through a single midline incision, avoiding the need for separate incisions on each side. However, this technique demands high surgical expertise due to its intricate nature and requires meticulous reconstruction steps. Surgeons performing the Rives-Stoppa repair must have extensive experience to achieve optimal results, as it necessitates a longer surgical duration compared to the Lichtenstein technique [23]. In contrast, bilateral inguinal hernia repair using the Lichtenstein tension-free mesh repair technique involves placing a synthetic mesh over the posterior inguinal wall on each side through separate incisions. The mesh is secured without tension to reinforce the weakened areas and prevent recurrence. This approach is generally less technically demanding than the Rives-Stoppa repair, as it involves placing the mesh over the inguinal wall rather than extensive dissection and reconstruction [24].

Operating Time

The operating time for the Rives-Stoppa repair and other related procedures varies across different studies. One study reported the mean operation time for the Rives-Stoppa repair as 103.3 minutes, ranging from 85 to 145 minutes. Another study found a longer mean operative time of 170.47 ± 15.08 minutes for the Rives-Stoppa repair [25]. When comparing the Rives-Stoppa repair to the Transversus Abdominis Release (TAR) procedure, the TAR approach took longer, with a mean operative time of 188.8 ± 22.04 minutes. This suggests that the Rives-Stoppa repair may be a more time-efficient technique [26]. Progrip self-gripping mesh in Rives-Stoppa repair has been shown to reduce operative time further. One study found that the mean operative time for the Progrip group was 101 ± 29.5 minutes, significantly shorter than the non-progrip group, which had a mean of 121 ± 39.8 minutes [27]. The operating time for the Rives-Stoppa repair typically ranges from approximately 85 to 145 minutes, with a mean of around 103.3 minutes. The TAR procedure takes longer, with a mean of about 188.8 minutes. However, using Progrip self-gripping mesh can reduce the operative time for the Rives-Stoppa repair compared to traditional mesh fixation methods [26].

Use of Mesh and Materials

The selection of mesh and materials for Rives-Stoppa repair and bilateral inguinal hernioplasty is crucial. In Rives-Stoppa repair, large prosthetic meshes are commonly used to reinforce the entire abdominal wall. Research has shown favorable outcomes using polyvinylidene fluoride (PVDF) meshes like DynaMesh®-CICAT or DynaMesh®-IPOM (DynaMesh, Germany), with reported low recurrence and complication rates [28]. Another study compared a self-gripping Progrip mesh in Rives-Stoppa repair, noting reduced postoperative pain and lower hematoma rates compared to polypropylene mesh secured with sutures [28]. In contrast, the Lichtenstein bilateral inguinal hernia repair technique typically involves placing synthetic mesh over the posterior inguinal wall on both sides. Surgeons often choose materials such as polypropylene based on personal preference. Both Rives-Stoppa repair and the Lichtenstein technique utilize synthetic meshes, but the specific type and fixation method selected can significantly influence outcomes such as postoperative pain and complication rates [27].

Intraoperative Complications

The Rives-Stoppa repair technique, especially when employing self-gripping meshes, has been associated with various intraoperative complications. One significant concern is the formation of seromas. A comparative study between retromuscular Progrip self-gripping mesh and traditional polypropylene mesh fixed with sutures revealed fewer seromas in the Progrip group, with only two patients affected compared to three in the non-Progrip group [27]. Another reported complication during Rives-Stoppa repair is a hematoma. In the same study, the Progrip mesh group experienced no hematomas, whereas four patients in the non-progrip group developed hematomas [27]. Wound infection is also a potential complication associated with the Rives-Stoppa technique. Research focusing on ventral incisional hernia repair using the Rives-Stoppa method indicated a low overall recurrence rate. Still, it highlighted the risk of prosthetic infection necessitating mesh removal, which could contribute to hernia recurrence [13]. Additionally, a study investigating a modified Rives-Stoppa technique using composite mesh reported early complications such as seroma (16.3%), hematoma (11.6%), wound infection (7.0%), and rare instances of bowel injury (2.3%) [29]. These findings underscore the importance of meticulous surgical technique and suggest potential benefits of utilizing self-gripping meshes in Rives-Stoppa repair to mitigate certain intraoperative complications.

Patient outcomes

Postoperative Recovery

Patients undergoing the Rives-Stoppa repair for incisional hernias generally experience manageable levels of postoperative pain and discomfort. Comparative studies have shown no significant differences in postoperative pain between the Rives-Stoppa repair and other techniques, such as the Lichtenstein method for inguinal hernias. Effective pain management strategies, including multimodal analgesia, are crucial in minimizing discomfort during recovery [13]. Regarding hospital stays, patients undergoing the Rives-Stoppa repair typically have shorter durations than other repair methods. For instance, research reported an average hospital stay of 6.5 days, while found that 80% of patients were discharged within eight days following the Rives-Stoppa repair. Compared to alternative approaches, this shorter hospitalization period is attributed to reduced morbidity and faster recovery times associated with the Rives-Stoppa technique [30]. It's important to consider individual patient factors such as underlying health conditions and the complexity of the hernia, as these can influence postoperative pain levels and length of hospital stay. Surgeons should carefully assess each patient's unique circumstances and tailor their treatment approach to optimize recovery outcomes [6].

Recurrence Rates

The Rives-Stoppa repair technique has shown outstanding outcomes with remarkably low recurrence rates for incisional hernias. According to a systematic review and meta-analysis, the recurrence rate following Rives-Stoppa (retro-rectus) repair after at least 24 months of follow-up was only 4.1% (95% CI: 3.0-5.6) [15]. Another study reported an even lower overall recurrence rate of 1.1% in patients undergoing the Rives-Stoppa repair for complex ventral incisional hernias, with an average follow-up of 96 months. When specifically examining studies including patients with recurrent incisional hernias and those undergoing their first hernia repair, the recurrence rate after at least 12 months was 3.7% (95% CI: 2.6-5.2) [31]. Comparatively, the Rives-Stoppa technique demonstrated a recurrence rate 3.7 times lower than that associated with onlay mesh repair. These findings underscore the efficacy of the Rives-Stoppa repair as a highly effective method for managing incisional hernias, offering consistently low recurrence rates even in long-term and complex cases. Such low recurrence rates position the Rives-Stoppa repair as a preferred option for addressing challenging abdominal wall defects [15].

Long-Term Complications

The Rives-Stoppa repair technique has shown excellent long-term outcomes in managing incisional hernias. Comparative studies indicate no significant differences in chronic pain between the Rives-Stoppa repair and other techniques like the Lichtenstein method for inguinal hernias. Patients undergoing the Rives-Stoppa repair commonly report good long-term outcomes with low chronic abdominal wall pain rates. This approach appears effective in minimizing chronic pain, with patients generally experiencing manageable levels of discomfort over time [13]. Regarding infection rates, the Rives-Stoppa repair is associated with relatively low rates of surgical site infections (SSIs), typically around 4%-5% in reviewed studies. Compared to techniques like IPOM repair, the Rives-Stoppa approach had a slightly higher rate of SSIs, with an odds ratio of 1.8 (95% CI: 1.03-3.14, p=0.038). However, the overall incidence of SSIs with the Rives-Stoppa repair remains relatively low, and careful surgical technique can effectively mitigate the risk of infectious complications. Prosthetic infections, potentially leading to hernia recurrence, were reported in approximately 3% of cases following Rives-Stoppa repair [32].

Quality of Life Assessments

The Rives-Stoppa repair technique has shown favorable outcomes in enhancing patients' quality of life following incisional hernia repair. Studies have indicated high levels of patient satisfaction and improved quality of life in the long-term with this approach. For instance, one study utilized the hernia-specific Carolina Comfort Scale (CCS) to assess the quality-of-life outcomes, revealing significant postoperative improvements with the Rives-Stoppa and TAR techniques. Interestingly, the Rives-Stoppa repair demonstrated superior CCS scores compared to TAR at postoperative day 90 [26]. Patients undergoing the Rives-Stoppa repair also experience manageable levels of postoperative pain and discomfort, with no significant differences in chronic groin pain compared to other techniques, such as the Lichtenstein method for inguinal hernias. This reduction in pain and discomfort contributes significantly to overall quality of life [13]. Additionally, the Rives-Stoppa approach facilitates a quicker return to normal activities and work, further contributing to patients' overall well-being. The excellent long-term outcomes and low recurrence rates associated with the Rives-Stoppa repair instill confidence in patients regarding the durability of results, further enhancing their quality of life [33].

Future directions

The future of surgical techniques is poised for significant advancements across multiple fronts. Innovations will continue to drive progress, particularly in minimally invasive surgery, which is expected to evolve further. This evolution will be supported by more adaptable and cost-effective surgical robots, potentially making robot-assisted surgery more accessible through increased portability. While robotic autonomy and machine learning advancements are anticipated, fully autonomous robots are not expected within the next two decades. Additionally, nano-robotics hold promise for applications in diagnosis and drug delivery [34]. Technological advancements in imaging, virtual reality (VR), and augmented reality (AR) will increasingly support surgical planning, training, and intraoperative guidance. Meanwhile, 3D printing will advance, enhancing capabilities in education, training, and preparation for complex surgical interventions. Materials science innovations will drive the development of novel implants, prosthetics, and tissue engineering scaffolds with enhanced biocompatibility and functionality. Smart materials that respond to physiological cues and deliver targeted therapies may revolutionize surgical devices and implants [35]. Looking beyond surgical techniques, the future may see significant strides in non-surgical interventions. Advances in genomics, big data analytics, and artificial intelligence are expected to pave the way for precision medicine, enabling more personalized and effective treatments tailored to individual patient characteristics. Non-surgical approaches, such as gene therapy, stem cell therapy, and targeted drug delivery, can potentially reduce the necessity for certain surgical procedures. However, challenges persist in ensuring equitable access to advanced surgical care, particularly in low- and middle-income countries. Addressing these disparities will require ongoing efforts to train more surgeons, develop affordable technologies, and establish robust healthcare infrastructure. These initiatives are crucial to ensuring that the benefits of technological advancements in surgery and medicine are accessible to all [36].

## Conclusions

In conclusion, the comparative analysis of the Rives-Stoppa repair and bilateral inguinal hernioplasty underscores the significance of choosing the appropriate surgical technique tailored to individual patient needs. Both methods demonstrate distinct advantages and challenges. The Rives-Stoppa repair is known for its effectiveness in complex and recurrent hernias, offering robust support through posterior mesh placement. On the other hand, bilateral inguinal hernioplasty, particularly in cases of bilateral hernias, provides a streamlined approach with potential benefits in terms of reduced operative time and quicker recovery. The review highlights that patient outcomes, including postoperative pain, recovery time, recurrence rates, and quality of life, can vary based on the technique employed. Furthermore, economic considerations such as the cost of surgery and overall cost-effectiveness play a crucial role in decision-making. As surgical technologies and materials evolve, ongoing research and clinical trials are essential to refine these techniques and improve patient outcomes. This comprehensive review aims to guide surgeons and healthcare providers in making informed decisions, ultimately enhancing the quality of care for patients with hernias.

## References

[1] Altomara D (2024). Altomara D: The basics of hernias. https://www.webmd.com/digestive-disorders/understanding-hernia-basics.

[2] (2024). Hernia: what it is, symptoms, types, causes & treatment. https://my.clevelandclinic.org/health/diseases/15757-hernia.

[3] (2024). Umbilical hernia: causes, symptoms, and treatments. https://www.medicalnewstoday.com/articles/189580.

[4] Jenkins JT, O'Dwyer PJ (2008). Inguinal hernias. BMJ.

[5] Coelho JC, Hajar FN, Moreira GA (2021). Femoral hernia: uncommon, but associated with potentially severe complications. Arq Bras Cir Dig.

[6] (2018). International guidelines for groin hernia management. Hernia.

[7] Fowler AJ, Trivedi B, Boomla K, Pearse R, Prowle J (2022). Change in healthcare utilisation after surgical treatment: observational study of routinely collected patient data from primary and secondary care. Br J Anaesth.

[8] Molina Caballero AY, Pérez Martínez A, Goñi Orayen C (2021). Abdominal hernia repair using the Rives-Stoppa technique: an abdominal reconstruction. Cir Pediatr.

[9] FitzGerald JF, Kumar AS (2014). Biologic versus synthetic mesh reinforcement: what are the pros and cons?. Clin Colon Rectal Surg.

[10] Rhemtulla IA, Fischer JP (2018). Retromuscular sublay technique for ventral hernia repair. Semin Plast Surg.

[11] Klinge U, Klosterhalfen B, Ottinger AP, Junge K, Schumpelick V (2002). PVDF as a new polymer for the construction of surgical meshes. Biomaterials.

[12] Notash AY, Notash AY Jr, Farshi JS, Amoli HA, Salimi J, Mamarabadi M (2007). Outcomes of the Rives-Stoppa technique in incisional hernia repair: ten years of experience. Hernia.

[13] Jain Y, Gianchandani Gyani SG, Chauhan S, Nayak K, Jain Y, Malhotra G, Rekavari SG (2024). Comparative analysis of bilateral open inguinal hernia repair and rives-stoppa repair: a comprehensive review. Cureus.

[14] Khansa I, Janis JE (2019). The 4 principles of complex abdominal wall reconstruction. Plast Reconstr Surg Glob Open.

[15] Hartog FP, Sneiders D, Darwish EF (2022). Favorable outcomes after retro-rectus (rives-stoppa) mesh repair as treatment for noncomplex ventral abdominal wall hernia, a systematic review and meta-analysis. Ann Surg.

[16] Xie J, Koo DC, Lee MJ, Sugiyama G (2024). The evolution of minimally invasive inguinal hernia repairs. Ann Laparosc Endosc Surg.

[17] Kelley WE Jr (2008). The evolution of laparoscopy and the revolution in surgery in the decade of the 1990s. JSLS.

[18] Fitzgibbons RJ Jr, Salerno GM, Filipi CJ, Hunter WJ, Watson P (1994). A laparoscopic intraperitoneal onlay mesh technique for the repair of an indirect inguinal hernia. Ann Surg.

[19] Maghsoudi H, Pourzand A (2005). Giant prosthetic reinforcement of the visceral sac: the Stoppa groin hernia repair in 234 patients. Ann Saudi Med.

[20] Open Inguinal Hernia Repair Technique (2024). Open inguinal hernia repair technique: approach considerations, Lichtenstein tension-free mesh repair, other approaches. https://emedicine.medscape.com/article/1534281-technique.

[21] Messaris E, Nicastri G, Dudrick SJ (2010). Total extraperitoneal laparoscopic inguinal hernia repair without mesh fixation: prospective study with 1-year follow-up results. Arch Surg.

[22] Chiow AKH, Chong CK, Tan S-M (2010). Inguinal hernias: a current review of an old problem. Proc Singap Healthc.

[23] Abdur Raheem J, Annu SC, Begum R, Iqbal H, Mohammad AM (2022). Defining wider indications for stoppa repair other than recurrent hernias. Cureus.

[24] Sakorafas GH, Halikias I, Nissotakis C, Kotsifopoulos N, Stavrou A, Antonopoulos C, Kassaras GA (2001). Open tension free repair of inguinal hernias; the Lichtenstein technique. BMC Surg.

[25] Li B, Qin C, Bittner R (2020). Endoscopic totally extraperitoneal approach (TEA) technique for primary ventral hernia repair. Surg Endosc.

[26] Kumar R, Prakash P, Sinha SR, Ahmad N, Baitha KS (2023). Short-term outcomes and quality-of-life assessment following rives-stoppa and transversus abdominis release procedures of open ventral hernia repair. Cureus.

[27] Bueno-Lledó J, Torregrosa A, Arguelles B, Carreño O, García P, Bonafé S, Iserte J (2017). Progrip self-gripping mesh in Rives-Stoppa repair: are there any differences in outcomes versus a retromuscular polypropylene mesh fixed with sutures? A “case series” study. Int J Surg Case Rep.

[28] Gossetti F, D’amore L, Grimaldi MR, Annesi E, Bambi L, Fernandez D, Negro P (2023). Rives-Stoppa repair of incisional hernias using PVDF mesh: a 10-year experience of a dedicated surgical team. J Sci Med Central.

[29] Fei L, Munegato G, Allaria A (2023). A modified Rives-Stoppa technique with composite mesh (FLaPp) in large incisional hernia: a multicentric retrospective cohort study. Eur Surg.

[30] Masati B, Haxhiu A, Dhima M, Punmira T, Zikaj G, Ibrahimi A, Dogjani A (2024). The benefit of open Rives-Stoppa procedure in complex incisional hernia. Albanian J Truma Emerg Surg.

[31] Rogmark P, Smedberg S, Montgomery A (2018). Long-term follow-up of retromuscular incisional hernia repairs: recurrence and quality of life. World J Surg.

[32] Pereira-Rodrigues AK, Maceio-Da-Graça JV, Ferreira EM, Alves-Almeida CC (2023). Onlay versus rives-stoppa techniques in the treatment of incisional hernias. Arq Bras Cir Dig.

[33] Bauer JJ, Harris MT, Gorfine SR, Kreel I (2002). Rives-Stoppa procedure for repair of large incisional hernias: experience with 57 patients. Hernia.

[34] Patel BJ: Council Post (2024). Council Post: The future of surgical robotics: innovations and predictions. https://www.forbes.com/sites/forbestechcouncil/2024/07/01/the-future-of-surgical-robotics-innovations-and-predictions/.

[35] Bui T, Ruiz-Cardozo MA, Dave HS (2024). Virtual, augmented, and mixed reality applications for surgical rehearsal, operative execution, and patient education in spine surgery: a scoping review. Medicina (Kaunas).

[36] Johnson KB, Wei WQ, Weeraratne D (2021). Precision medicine, AI, and the future of personalized health care. Clin Transl Sci.

